# Estimating CDMs Using the Slice-Within-Gibbs Sampler

**DOI:** 10.3389/fpsyg.2020.02260

**Published:** 2020-09-25

**Authors:** Xin Xu, Jimmy de la Torre, Jiwei Zhang, Jinxin Guo, Ningzhong Shi

**Affiliations:** ^1^Key Laboratory of Applied Statistics of MOE, School of Mathematics and Statistics, Northeast Normal University, Changchun, China; ^2^Faculty of Education, The University of Hong Kong, Pokfulam, Hong Kong; ^3^Key Lab of Statistical Modeling and Data Analysis of Yunnan Province, School of Mathematics and Statistics, Yunnan University, Kunming, China

**Keywords:** the slice-within-Gibbs sampler, CDMs, DINA model, G-DINA model, Gibbs sampling, MH algorithm

## Abstract

In this paper, the slice-within-Gibbs sampler has been introduced as a method for estimating cognitive diagnosis models (CDMs). Compared with other Bayesian methods, the slice-within-Gibbs sampler can employ a wide-range of prior specifications; moreover, it can also be applied to complex CDMs with the aid of auxiliary variables, especially when applying different identifiability constraints. To evaluate its performances, two simulation studies were conducted. The first study confirmed the viability of the slice-within-Gibbs sampler in estimating CDMs, mainly including G-DINA and DINA models. The second study compared the slice-within-Gibbs sampler with other commonly used Markov Chain Monte Carlo algorithms, and the results showed that the slice-within-Gibbs sampler converged much faster than the Metropolis-Hastings algorithm and more flexible than the Gibbs sampling in choosing the distributions of priors. Finally, a fraction subtraction dataset was analyzed to illustrate the use of the slice-within-Gibbs sampler in the context of CDMs.

## 1. Introduction

Cognitive diagnosis models (CDMs) aim to provide a finer-grained evaluation of examinees' attribute profiles. As psychometric tools, CDMs have been employed in both educational and non-educational contexts (Rupp and Templin, 2008; de la Torre et al., 2018). Thus far, several reduced and general CDMs have been proposed. Examples of the former are the *deterministic inputs, noisy* “*and*” *gate* (DINA; Junker and Sijtsma, 2001) model and *deterministic inputs, noisy* “*or*” *gate* (DINO; Templin and Henson, 2006) model; whereas examples of the latter are the generalized DINA (G-DINA; de la Torre, 2011) model, *log-linear* CDM (Henson et al., 2009), and *general diagnostic* model (GDM; von Davier, 2008). When applying CDMs, a fundamental issue is model identifiability of the Q-matrix. For different models, different identifiability conditions have been proposed, including strict identifiability (Liu et al., 2013; Chen et al., 2015; Xu, 2017) and milder identifiability (Chen et al., 2020; Gu and Xu, 2020).

Basically, in the CDM literature, two estimation methods were widely used. The first is the Expectation-Maximization (EM) algorithm within the frequentist framework (de la Torre, 2009, 2011; Huo and de la Torre, 2014; Chiu et al., 2016; George et al., 2016; Minchen et al., 2017; Kuo et al., 2018). However, the main motivation of CDMs is to identify the latent attribute profiles of examinees' and Bayesian methods are often more natural to reach the goal. The second most commonly used method is Markov chain Monte Carlo (MCMC) method (de la Torre and Douglas, 2004; Culpepper, 2015; Culpepper and Hudson, 2018; Zhan et al., 2018, 2019; Jiang and Carter, 2019). Usually, to use the MH algorithm, it is necessary to choose a proposal distribution that can lead to optimal sampling efficiency. However, empirically determining the optimal proposal distribution can be challenging in practice. Culpepper (2015) first introduced the Gibbs sampling to the DINA model and Zhang et al. (2020) applied the Pólya-Gamma Gibbs sampling based on auxiliary variables to DINA model. Culpepper and Hudson (2018) introduced Bayesian method to the Reduced Reparameterized Unified Model (rRUM; DiBello et al., 1995; Roussos et al., 2007).

With the development of the identifiability, more complex restrictions need to be taken into account. How to estimate more general models incorporating to the corresponding identifiability conditions has been a technically appealing task. In this paper, a sampling method called the slice-within-Gibbs sampler is introduced, in which the identifiability constraints are easy to be imposed. The slice-within-Gibbs sampler can avoid the boring choices of tunning parameters in the MH algorithm and converges faster over the MH algorithm with misspecified proposal distributions. In addition, it has more flexibility over the Gibbs sampling in prior choices and can be easier to apply to more general models compared with the Pólya-Gamma Gibbs Sampling and the Gibbs sampling. In line with the original idea of the slice-within-Gibbs sampler, data would still be augmented with auxiliary variables to make sampling from complicated posterior densities feasible. Existing theoretical results on convergence and stability of the slice-within-Gibbs sampler guarantees that the method is equally applicable to psychometric models, in general, and CDMs, in particular. As such, this paper focuses mainly on demonstrating the usage, as well as evaluating the performance of the slice-within-Gibbs sampler in conjunction with CDMs.

The remainder of this paper is organized as follows. Section 2 provides an overview of CDMs, mainly the G-DINA and DINA models. A detailed slice sampler algorithm for the DINA model is presented in section 3, followed by some advantages of the algorithm. In section 4, two simulations are conducted to illustrate the feasibility of the sampler and its advantages over other MCMC methods. Section 5 contains an application of the slice-within-Gibbs sampler to fraction subtraction data, and section 6 provides a discussion of the findings and limitations of this work and possible future research directions.

## 2. Overview of CDMs

Suppose there are a total of *I* examinees and *J* items with *K* required attributes in a test. Let *Y*_*ij*_ denote the binary response of examinee *i* to item *j*, and ***Y*** = {*Y*_*ij*_}_*I*×*J*_ be the response matrix. In CDMs, it is often assumed that the latent trait of examinees is quantified by *K*−dimensional vectors, called attribute profiles. That is, for *i*th examinee, the latent profile is ***α***_***i***_ = (α_*i*1_, α_*i*2_, …, α_*iK*_), where α_*ik*_ ∈ {0, 1} and α_*ik*_ = 1 means that examinee *i* has mastered the *k*th attribute, whereas α_*ik*_ = 0 otherwise. Therefore, there possibly exist *C* = 2^*K*^ different attribute profile classes, denoted by ***α***_***c***_ = (α_*c*1_, α_*c*2_, …, α_*cK*_), *c* = 1, 2, …, *C*. The association between items and attributes is specified by Q-matrix Q = {_*q*_*jk*__}_*J*×*K*_ (Tatsuoka, 1983), where *q*_*jk*_ = 1 means the *k*th attribute is required to answer *j*th item correctly, and *q*_*jk*_ = 0 otherwise.

CDMs model the item response *Y*_*ij*_ using the following Bernoulli distribution,

(1) $$\begin{array}{l} P\left(Y_{ij}=y_{ij}|\alpha_{i},\Omega_{j}\right)=f_{ij}^{y_{ij}}h_{ij}^{1-y_{ij}}, \end{array}$$

where *f*_*ij*_ = 1 − *h*_*ij*_ = *P*(*Y*_*ij*_ = 1|***α***_*i*_ = ***α***_*c*_, Ω_*j*_) is the probability of answering item *j* correctly for examinee *i* with attribute pattern ***α***_***c***_, and Ω_*j*_ denotes the unknown parameter set of item *j*. The likelihood of the data can be written by obtaining the weighted sum across the different attribute profiles. More specifically, assuming an identically and independently distributed latent membership, π_*c*_ = *P*(***α***_***i***_ = ***α***_***c***_), the joint likelihood can be written as,

(2) $$\begin{array}{l} P\left(Y|\Omega ,\pi \right)=\overset{I}{\underset{i=1}{\prod }}\overset{J}{\underset{j=1}{\prod }}\overset{C}{\underset{c=1}{\sum }}\pi_{c}P\left(Y_{ij}=y_{ij}|\alpha_{i}=\alpha_{c},\Omega_{j}\right). \end{array}$$

### 2.1. The G-DINA Model

The G-DINA model is a saturated CDM that subsumes a number of reduced CDMs. In this model, *P*(*Y*_*ij*_ = 1|***α***_*i*_, Ω_*j*_) in Equation (1) is expressed as a function of the main effects and interactions of the required attributes for each item. Following de la Torre (2011), let $K_{j}^{*}=\overset{K}{\underset{k=1}{\sum }}q_{jk}$ denote the number of required attributes for item *j*. For notational convenience, but without loss of generality, let the first $K_{j}^{*}$ attributes be required for item *j*, and let $\alpha_{ij}^{*}=\left(\alpha_{i1},\alpha_{i2},\cdots \;,\alpha_{iK_{j}^{*}}\right)$ be the reduced vector of ***α***_*i*_ associated with item *j*. The *f*_*ij*_ in the G-DINA model for item *j* is,

(3) $$\begin{array}{l} P\left(Y_{ij}=1|\alpha_{ij}^{*},\delta_{j}\right)=\delta_{j0}+\overset{K_{j}^{*}}{\underset{k=1}{\sum }}\delta_{jk}\alpha_{ik}\\ \;+\overset{K_{j}^{*}}{\underset{k^{\prime }=k+1}{\sum }}\overset{K_{j}^{*}-1}{\underset{k=1}{\sum }}\delta_{jkk^{\prime }}\alpha_{ik}\alpha_{ik^{\prime }}\cdots +\delta_{j12\cdots K_{j}^{*}}\overset{K_{j}^{*}}{\underset{k=1}{\prod }}\alpha_{ik}, \end{array}$$

where δ_*j*0_ is the intercept; δ_*jk*_ is the main effect of α_*ik*_; $\delta_{jkk^{\prime }}$ is the two-way interaction effect of α_*ik*_ and $\alpha_{ik^{\prime }}$; and $\delta_{j12\cdots K_{j}^{*}}$ is the $K_{j}^{*}$-way interaction effect of $\alpha_{i1},\cdots \;,\alpha_{iK_{j}^{*}}$. Aside from the identity link, the G-DINA model can be expressed using log and logit links (de la Torre, 2011).

### 2.2. The DINA Model

The DINA model is one of most commonly used CDMs, and its *f*_*ij*_ is given by

(4) $$P\left(Y_{ij}=1|\alpha_{ij}^{*},s_{j},g_{j}\right)=\{\begin{array}{ll} 1-s_{j} & \text{for }\alpha_{\text{ij}}^{*}\text{=}1,\\ g_{j} & \text{otherwise,} \end{array}$$

where *g*_*j*_ and *s*_*j*_ are the guessing and slip parameters, and $\alpha_{ij}^{*}=1={\left(1,1,\ldots ,1\right)}^{T}$ denotes that examinee *i* has possessed all the required attributes of item *j*. In the DINA model, Ω_*j*_ = {*g*_*j*_, *s*_*j*_}.

As many researchers have already noted, the DINA model is a special case of the G-DINA model. The former can be derived from the latter by setting δ_*j*0_ = *g*_*j*_, $\delta_{j0}+\delta_{j12\cdots \;,K_{j}^{*}}=1-s_{j}$, and remaining parameters to zero. Thus, in the DINA model, only the $K_{j}^{*}$-way interaction is taken into account, which indicates that the response is expected to be correct only when all the required attributes have been mastered.

### 2.3. Identifiability of Restricted Latent Class Models

For most common statistical inferences, the identifiability of the models is a precondition. To guarantee the identifiability when estimating CDMs, we follow a set of sufficient conditions presented by Xu (2017) for a class of restricted latent class models. Specifically, these CDMs need to satisfy the following assumptions:

(*i*) (Monotonicity relations) For any attribute profile ${\alpha^{\prime }}_{i}$,

(5) $$\begin{array}{l} P(Y_{ij}=1|\alpha_{i},\Omega_{j},\alpha_{i}≽q_{j})\geq P(Y_{ij}=1|{\alpha^{\prime }}_{i},\Omega_{j})\\ \;\geq P(Y_{ij}=1|\alpha_{i},\Omega_{j},\alpha_{i}=0); \end{array}$$

and (*ii*) If ***q***_*j*_ = ***e***_*k*_ for *k* = 1, 2, …, *K*,

(6) $$\begin{array}{l} P\left(Y_{ij}=1|\alpha_{i},\Omega_{j},\alpha_{i}=1\right)>P\left(Y_{ij}=1|\alpha_{i},\Omega_{j},\alpha_{i}⋡e_{k}\right), \end{array}$$

where ***α*** ≽ ***q*** holds if and only if α_*k*_ ≥ *q*_*k*_ for any *k* ∈ {1, 2, …, *K*} and ***α*** ⋡ ***q*** means there exists at least one *k* ∈ {1, 2, …, *K*} such that α_*k*_ < *q*_*k*_; **0** = (0, 0, …, 0)^*T*^; and ***e*_*k*_** is a vector whose *k*th element is one and the rest elements are zero.

Both the G-DINA and DINA models are considered restricted latent class models. Specifically, for the DINA model, the above assumptions are equivalent to 1−*s*_*j*_ > *g*_*j*_. For the G-DINA model, the transformation is more complicated and will be discussed in section 3.2.

Identifiability in restricted latent class models satisfies the following sufficient conditions (Xu, 2017):

(C1) The Q-matrix is constructed such that

$$Q=\left(\begin{array}{c} I_{K}\\ I_{K}\\ Q^{\prime } \end{array}\right),$$

where *I*_*K*_ is a *K* × *K* identity matrix; and

(C2) For any item in *Q*′, examinees who possess no required attributes have the lowest success probabilities. That is, $\underset{\alpha_{i}\neq 0}{\min}P\left(Y_{ij}=1|\alpha_{i},\Omega_{j}\right)>P\left(Y_{ij}=1|\alpha_{i}=0,\Omega_{j}\right)$, for *j* > 2*K*.

## 3. Introducing the Slice-Within-Gibbs Sampler for CDMs

In this section, we introduce the slice-within-Gibbs sampler as a method of estimating CDMs. Moreover, we list its advantages.

### 3.1. Using the Slice-Within-Gibbs Sampler to Estimate CDMs

First, the joint posterior distribution of model parameters (***Ω**, **π***) could be written as,

(7) $$\begin{array}{l} P\left(\Omega ,\pi |Y\right)\propto P\left(Y|\Omega ,\pi \right)P\left(\Omega ,\pi \right), \end{array}$$

where *P*(***Ω**, **π***) denotes the joint prior distribution.

Step 1: Sample the positive auxiliary variables *U*_*ij*_ and *V*_*ij*_ from the following posterior distribution,

(8) $$\begin{array}{l} U_{ij}|Y,\Omega ,\pi \sim \text{Uniform}\left(0,f_{ij}\right),\text{if }Y_{ij}=1,\\ V_{ij}|Y,\Omega ,\pi \sim \text{Uniform}\left(0,h_{ij}\right),\text{if }Y_{ij}=0. \end{array}$$

The joint posterior distribution *P*(***Ω, π***, ***U***, ***V***|***Y***) is proportional to

$$\begin{array}{l} \overset{I}{\underset{i=1}{\prod }}\overset{J}{\underset{j=1}{\prod }}\left[{\text{I}}_{\left(Y_{ij}=1\right)}\left(Y_{ij}\right){\text{I}}_{\left(0,f_{ij}\right)}\left(U_{ij}\right)+{\text{I}}_{\left(Y_{ij}=0\right)}\left(Y_{ij}\right){\text{I}}_{\left(0,h_{ij}\right)}\left(V_{ij}\right)\right]P\left(\Omega ,\pi \right). \end{array}$$

Note that $P\left(\Omega ,\pi |Y\right)=\int_{0}^{\infty }\int_{0}^{\infty }P\left(\Omega ,\pi ,U,V|Y\right)dUdV$, which means that considering the above posterior distribution is enough to estimate (***Ω**, **π***); I(·) denotes the indicator function, and I_(_*Y*__*ij*_ = 1)_(*Y*_*ij*_) = 1 if *Y*_*ij*_ = 1, and I_(_*Y*__*ij*_ = 1)_(*Y*_*ij*_) = 0 otherwise.

Step 2: Sample item parameters Ω_*j*_, *j* = 1, 2, …, *J*, from the following truncated distribution:

(9) $$\begin{array}{l} \Omega_{j}|Y,U,V,\Omega_{-j},\pi \sim P\left(\Omega_{j}\right){\text{I}}_{\left(\Omega_{j}^{L}<\Omega_{j}<\Omega_{j}^{R}\right)}\left(\Omega_{j}\right), \end{array}$$

where $\Omega_{j}^{L}$ and $\Omega_{j}^{R}$ are derived from the identifiability restrictions, and inequalities 0 < *U*_*ij*_ < *f*_*ij*_, and 0 < *V*_*ij*_ < *h*_*ij*_. For example, in the DINA model, $s_{j}^{L}=\max\left\{\underset{i\in {▽}_{j}}{\max}\left\{V_{ij}\right\},0\;\right\}$, and $s_{j}^{R}=\min\left\{\underset{i\in {△}_{j}}{\min}\left\{1-U_{ij}\right\},1-g_{j}\right\}\;$, where ${▽}_{j}=\left\{i|Y_{ij}=0,\alpha_{ij}^{*}=1\right\}$ and ${△}_{j}=\left\{i|Y_{ij}=1,\alpha_{ij}^{*}=1\right\}$. In the G-DINA model,

$$\begin{array}{l} \delta_{jk}^{L}=\max\left\{\underset{i\in F_{j}}{\max}\left\{U_{ij}-\left(\delta_{j0}+\overset{K_{j}^{*}}{\underset{k^{\prime }=k+1}{\sum }}\overset{K_{j}^{*}-1}{\underset{k=1}{\sum }}\delta_{jkk^{\prime }}\alpha_{ik}\alpha_{ik^{\prime }}\cdots +\delta_{j12\cdots K_{j}^{*}}\overset{K_{j}^{*}}{\underset{k=1}{\prod }}\alpha_{ik}\right)\right\},\delta^{*L}\right\},\text{and}\\ \delta_{jk}^{R}=\min\left\{\underset{i\in \Pi_{j}}{\min}\left\{1-V_{ij}-\left(\delta_{j0}+\overset{K_{j}^{*}}{\underset{k^{\prime }=k+1}{\sum }}\overset{K_{j}^{*}-1}{\underset{k=1}{\sum }}\delta_{jkk^{\prime }}\alpha_{ik}\alpha_{ik^{\prime }}\cdots +\delta_{j12\cdots K_{j}^{*}}\overset{K_{j}^{*}}{\underset{k=1}{\prod }}\alpha_{ik}\right)\right\},\delta^{*R},1-\underset{-jk}{\sum }\delta_{j}\right\}, \end{array}$$

where Π_*j*_ = {*i*|*Y*_*ij*_ = 0}, *F*_*j*_ = {*i*|*Y*_*ij*_ = 1}, and δ^*L^ and δ^*R^ are the lower and upper bounds, respectively, determined from the identifiability conditions of the restricted models.

Step 3: Update the latent membership probabilities ***π*** and the latent profile ***α***_***i***_. Following Huebner and Wang (2011) and Culpepper (2015), the prior of ***π*** is assumed to follow Dirichlet(φ_0_, …, φ_0_). The full conditional distribution of the latent class probabilities ***π*** can be written as

$$\begin{array}{l} \pi |\alpha_{1},\ldots ,\alpha_{C}\sim \text{Dirichlet}(\varphi_{0}\\ \;+\sum_{i=1}^{I}{\text{I}}_{\left(\alpha_{i}=\alpha_{1}\right)}(\alpha_{i}),\ldots ,\varphi_{0}+\sum_{i=1}^{I}{\text{I}}_{\left(\alpha_{i}=\alpha_{1}\right)}(\alpha_{i})). \end{array}$$

In this process, ***α***_***i***_ is sampled from the distribution

(10) $$\begin{array}{l} \alpha_{i}|Y_{i},s,g,\pi \sim \text{Multinomial}\left(1,\left[\varsigma_{i1},...,\varsigma_{iC}\right]\right), \end{array}$$

where

$$\varsigma_{ic}=P\left(\alpha_{i}=\alpha_{c}|Y_{i},\Omega ,\pi \right)=\frac{\pi_{c}\prod_{j=1}^{J}P\left(Y_{ij}|\alpha_{i}=\alpha_{c},\Omega_{j}\right)}{\overset{C}{\underset{c=1}{\sum }}\pi_{c}\prod_{j=1}^{J}P\left(Y_{ij}|\alpha_{i}=\alpha_{c},\Omega_{j}\right)}.$$

A number of differences exist in updating the item parameters using the MH algorithm, Gibbs sampling, and slice-within-Gibbs sampler. The MH algorithm samples the new value from a proposal distribution *p*_*proposal*_(Ω_*j*_). In this paper, we adopted truncated normal distributions as the proposal distributions. Within the Gibbs sampling framework, samples are drawn from the posterior distributions, which is a feature inherited by the slice-within-Gibbs sampler. For practicability, conjugate priors are normally employed for in the Gibbs sampling. In the “dina” 3 R package, for example, Culpepper (2015) used the Gibbs sampler to estimate the DINA model. In contrast, to make sampling more convenient and flexible to implement, the slice-within-Gibbs sampler transforms the posterior distributions of item parameters into a uniform distribution by introducing auxiliary variables. However, for updating the latent membership probabilities ***π*** and the latent profile ***α***_***i***_, the same formula was adopted by all the samplers.

### 3.2. About the Monotonicity Restrictions

When applying the slice-within-Gibbs sampler, the monotonicity restrictions are needed to cooperate with Step 3 for identifiability. For DINA model, it is easy to implement this constraint, that is, *s*_*j*_ + *g*_*j*_ < 1. However for other complex CDMs, it is a bit complicated. In this part, we present how to restrict parameters specially in G-DINA model.

In this part, we only took *K* = 3 as an example. When *K* = 3, there exist at most 2^*K*^ = 8 parameters and corresponding *C* classes in G-DINA model. The inequalities (5) and (6) are actually equivalent to adopt the following inequality considering all combinations of the *q*−entries.

(11) $$\begin{array}{l} 0\leq \delta_{0}\leq \begin{array}{c} \delta_{0}+\delta_{1}\\ \delta_{0}+\delta_{2}\\ \delta_{0}+\delta_{3} \end{array}\leq \begin{array}{c} \delta_{0}+\delta_{1}+\delta_{2}+\delta_{12}\\ \delta_{0}+\delta_{1}+\delta_{3}+\delta_{13}\\ \delta_{0}+\delta_{2}+\delta_{3}+\delta_{23} \end{array}\leq \begin{array}{c} \delta_{0}+\delta_{1}+\delta_{2}+\delta_{3}\\ +\delta_{12}+\delta_{13}+\delta_{23}+\delta_{123}\leq 1. \end{array} \end{array}$$

Therefore, the corresponding bound can be imposed as follows:

1. Consider $\delta_{0}^{\left(t+1\right)}\in \left[\delta_{0}^{L},\delta_{0}^{R}\right]$ and $\delta_{0}^{L}=0$, $\delta_{0}^{R}=\min\left\{P\left(1,0,0\right),P\left(0,1,0\right),P\left(0,0,1\right)\right\}$, where *P*(***α***) = *P*(*Y*_*ij*_ = 1|***α***).

2. Consider $\delta_{1}^{\left(t+1\right)}$ which is equivalent to consider $\delta^{*}=\delta_{1}^{\left(t+1\right)}+\delta_{0}^{\left(t+1\right)}$ and δ^*^ ∈ [δ^*L^, δ^*R^]. And

$$\{\begin{array}{l} \delta^{*L}=\delta_{0}^{\left(t+1\right)},\\ \delta^{*R}=\min\{P(1,1,0),P(1,0,1)\}. \end{array}$$

3. Apply similar formula to other main-effect parameters.

4. Consider $\delta_{12}^{\left(t+1\right)}$ which is equivalent to consider $\delta^{*}=\delta_{0}^{\left(t+1\right)}+\delta_{1}^{\left(t+1\right)}+\delta_{2}^{\left(t+1\right)}+\delta_{12}^{\left(t+1\right)}$ and δ^*^ ∈ [δ^*L^, δ^*R^]. And

$$\{\begin{array}{l} \delta^{*L}=\max\left\{P\left(1,0,0\right),P\left(0,1,0\right)\right\},\\ \delta^{*R}=P\left(1,1,1\right). \end{array}$$

### 3.3. Some Advantages of the Slice-Within-Gibbs Sampler

The MH algorithm typically relies heavily on the proposal distributions to achieve sampling efficiency. Under unidimensional cases, some researchers suggest that about 50% of candidates need to be accepted for an appropriate proposal distribution to be optimal. The probability of acceptance reduces to around 25% when sampling two- or three-dimensional parameters (Patz and Junker, 1999). For more complex CDMs, this probability needs to drop even more. Compared with MH algorithm, the slice-within-Gibbs sampler as an extension of the Gibbs sampler inherits the high efficiency of the latter. Specifically, the slice-within-Gibbs sampler avoids choosing a proposal distribution because the posterior acts as its proposal distribution. This gives the slice-within-Gibbs sampler acceptance probabilities equal to 1, which makes it highly efficient.

In contrast to the Gibbs sampler, the slice-within-Gibbs sampler has greater flexibility in choosing the prior distributions. Although highly efficient, finding easy-to-use conjugate prior distributions renders the use of the Gibbs sampler challenging in practice. However, this is not an issue with the slice-within-Gibbs sampler - its efficiency is not affected by the choice of prior distributions. Even if misspecified priors are adopted, it can obtain satisfactory results.

Thus, the slice-within-Gibbs sampler not only has a relatively high convergence rate, but also overcomes the dependence on the conjugate prior. Moreover, based on Theorem 7 in Mira and Tierney (2002), it can easily be shown that the slice-within-Gibbs sampler when used with CDMs is uniformly ergodic because *f*_*ij*_ is bounded by 1. However, it should be noted that a few other MCMC algorithms exhibit this robust property (Mira and Tierney, 1997; Roberts and Rosenthal, 1999).

## 4. Simulation Study

In this section, two simulation studies were conducted to evaluate the performance of the slice-within-Gibbs sampler in the CDM context. Simulation 1 was designed mainly to examine the extent the slice-within-Gibbs sampler can accurately recover the parameters of the DINA model and G-DINA models; Simulation 2 was designed to document the advantages of the slice-within-Gibbs sampler over the MH algorithm and Gibbs sampling in estimating the DINA model.

### 4.1. Simulation Study 1

#### 4.1.1. Design

**Figure 1:**
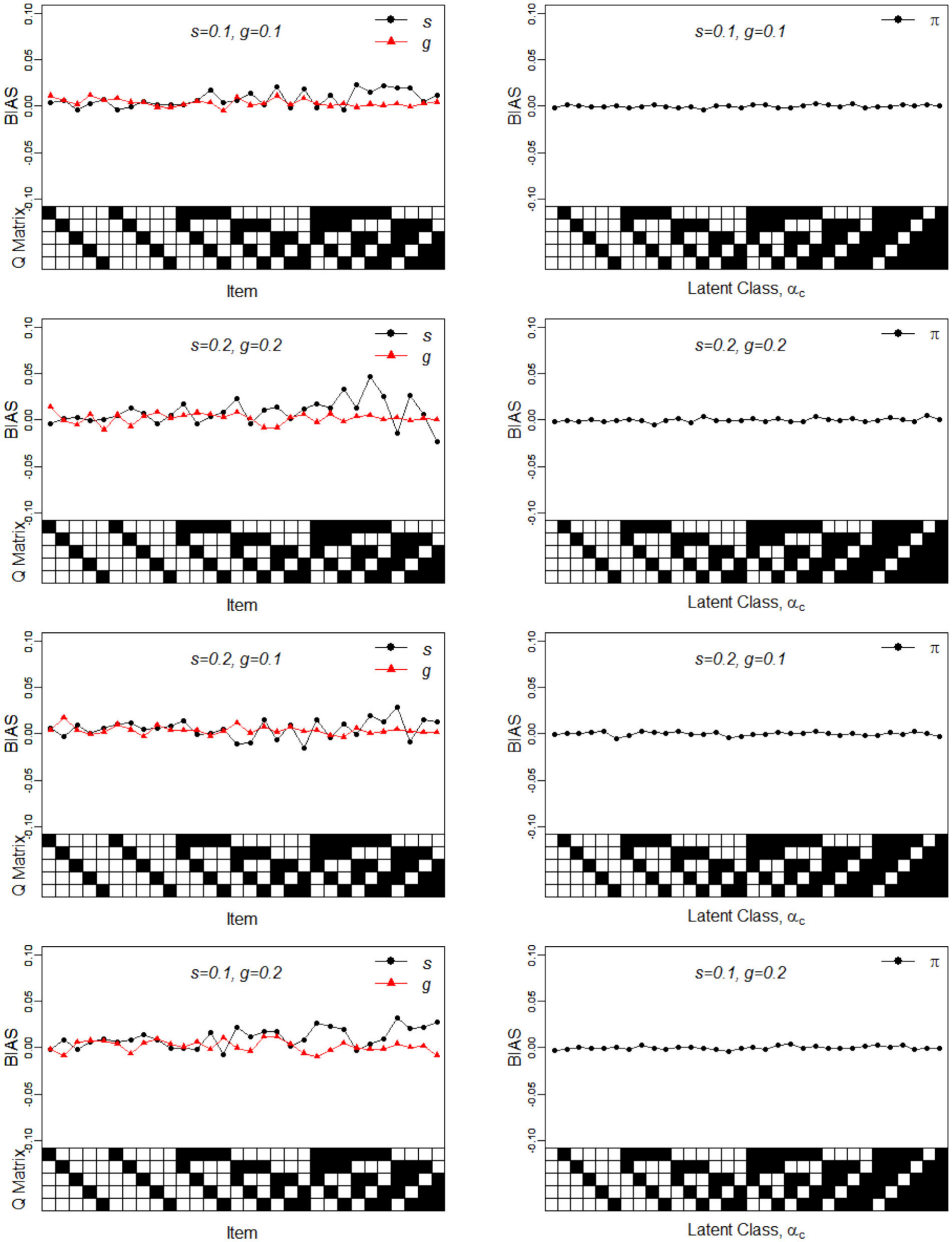
Bias of the slip, guessing, and the latent membership parameters under four different noise levels. Given on the X-axis are the Q-matrix and ***α***_***c***_, where a black square denotes the presence of the attribute.

**Figure 2:**
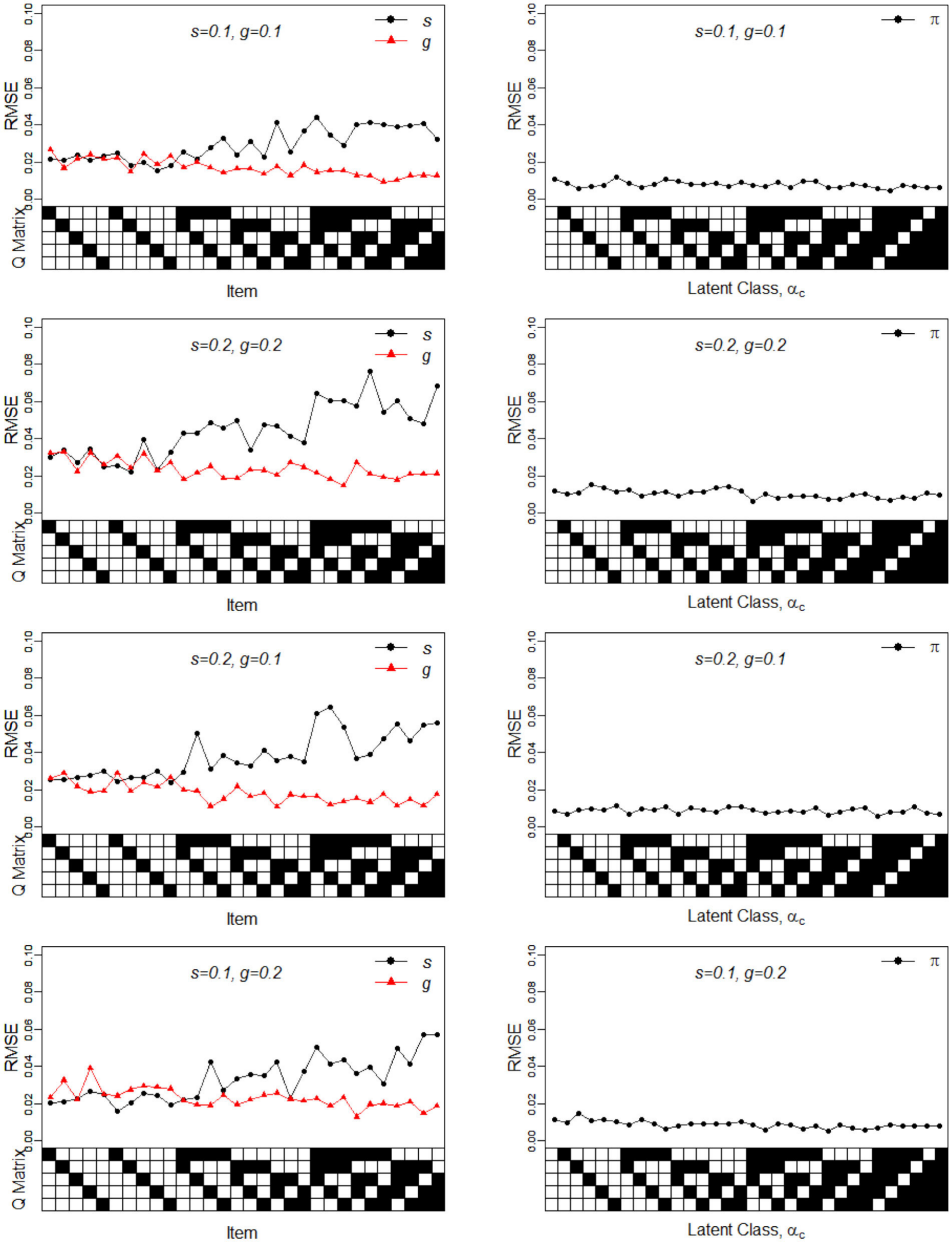
RMSE of the slip, guessing, and the latent membership parameters under four different noise levels. Given on the X-axis are the Q-matrix and ***α***_***c***_, where a black square denotes the presence of the attribute.

In Simulation Study 1, the number of attributes for the DINA and G-DINA models was fixed to *K* = 5 and *K* = 3, respectively, whereas the number of items was set to *J* = 30. The Q-matrices for the DINA model given in Figures 1, 2 and for the G-DINA model given in Table 1 were designed to ensure the identifiability of restricted latent class models.

**Table 1:** True Q-matrix for *K* = 3.

**Item**	**α_1_**	**α_2_**	**α_3_**	**Item**	**α_1_**	**α_2_**	**α_3_**
1	1	0	0	16	1	1	0
2	0	1	0	17	1	0	1
3	0	0	1	18	0	1	1
4	1	0	0	19	1	1	0
5	0	1	0	20	1	0	1
6	0	0	1	21	0	1	1
7	1	0	0	22	1	1	0
8	0	1	0	23	1	0	1
9	0	0	1	24	0	1	1
10	1	0	0	25	1	1	1
11	0	1	0	26	1	1	1
12	0	0	1	27	1	1	1
13	1	1	0	28	1	1	1
14	1	0	1	29	1	1	1
15	0	1	1	30	1	1	1

In this context, the number of examinees *I* is set as 500, 1000, 3000. As for item parameters of DINA model, five different conditions were considered. Following Huebner and Wang (2011) and Culpepper (2015), four noise levels (i.e., item qualities) were considered: (1) a low noise level - *s*_*j*_ = *g*_*j*_ = 0.1; (2) a high noise level - *s*_*j*_ = *g*_*j*_ = 0.2; (3) the slip parameter was higher than the guessing parameter - *s*_*j*_ = 0.2, *g*_*j*_ = 0.1; and (4) the guessing parameter was higher than the slip parameter - *s*_*j*_ = 0.1, *g*_*j*_ = 0.2. A fifth condition was considered, where, as in Zhan et al. (2018), the negative correlation between the item parameters based on the empirical data was taken into account. Specifically, the guessing and slip parameters were generated from the following: $\left(\text{logit}(g_{j}),\text{logit}(s_{j}))\sim N(\left(\begin{array}{c} -2.564\\ -1.995 \end{array}\right),\left(\begin{array}{cc} 1.233 & -0.415\\ -0.415 & 0.571 \end{array}\right)\right)$. Under this distribution, the mean guessing and slip parameters were 0.096 and 0.103, respectively; the corresponding maxima were 0.365 and 0.484, respectively. The true parameters of the G-DINA model are listed in Table 2. Finally, the latent class membership probabilities ***π*** were set to be equal for the different latent classes.

**Table 2:** True parameters of the G-DINA model items.

	**δ_*j*0_**	**δ_*j*1_**				**δ_*j*0_**	**δ_*j*1_**						
	**δ_*j*0_**	**δ_*j*1_**	**δ_*j*2_**	**δ_*j*12_**		**δ_*j*0_**	**δ_*j*1_**	**δ_*j*2_**	**δ_*j*12_**				
**Item**					**Item**	**δ_*j*0_**	**δ_*j*1_**	**δ_*j*2_**	**δ_*j*3_**	**δ_*j*12_**	**δ_*j*13_**	**δ_*j*23_**	**δ_*j*123_**
1	0.10	0.70			16	0.20	0.10	0.15	0.40				
2	0.10	0.70			17	0.20	0.10	0.15	0.40				
3	0.10	0.70			18	0.20	0.30	0.30	-0.05				
4	0.10	0.70			19	0.20	0.30	0.30	-0.05				
5	0.10	0.70			20	0.20	0.30	0.30	-0.05				
6	0.10	0.70			21	0.20	0.20	0.20	0.00				
7	0.10	0.70			22	0.20	0.20	0.20	0.00				
8	0.10	0.70			23	0.20	0.20	0.20	0.00				
9	0.10	0.70			24	0.20	0.20	0.20	0.00				
10	0.10	0.70			25	0.20	0.10	0.10	0.10	0.05	-0.05	0.05	0.15
11	0.10	0.70			26	0.20	0.10	0.10	0.10	0.10	0.10	0.05	0.05
12	0.10	0.70			27	0.20	0.10	0.10	0.10	0.05	-0.05	0.05	0.15
13	0.20	0.10	0.15	0.40	28	0.20	0.10	0.10	0.10	0.05	-0.05	0.05	0.15
14	0.20	0.10	0.15	0.40	29	0.20	0.10	0.10	0.10	0.10	0.10	0.05	0.05
15	0.20	0.10	0.15	0.40	30	0.20	0.10	0.10	0.10	0.05	-0.05	0.05	0.15

In this simulation study, all priors were set to be non-informative. With respect to the item parameters, priors of the slip and guessing parameters to the DINA model were set to be Uniform(0, 1), whereas *P*(**δ**) ∝ 1 in the support set was assumed for the G-DINA model.

Two criteria were used to evaluate quality of the parameter recovery, namely, the bias and root mean squared error (RMSE) of ***s***, ***g***, **δ**, **π** across 25 replications. In both simulation studies, the slice-within-Gibbs sampler was iterated 20,000 times for each replication, where the first 10,000 iterations were discarded as burn-in.

To evaluate the convergence, four chains started at overdispersed starting values were run. The potential scale reduction factor (PSRF) $\hat{R}$ (Brooks and Gelman, 1998) was computed using the R package “coda”(Plummer et al., 2006). A value of $\hat{R}$ less than 1.1 (Brooks and Gelman, 1998) was used as the criterion for chain convergence.

#### 4.1.2. Results

**Figure 3:**
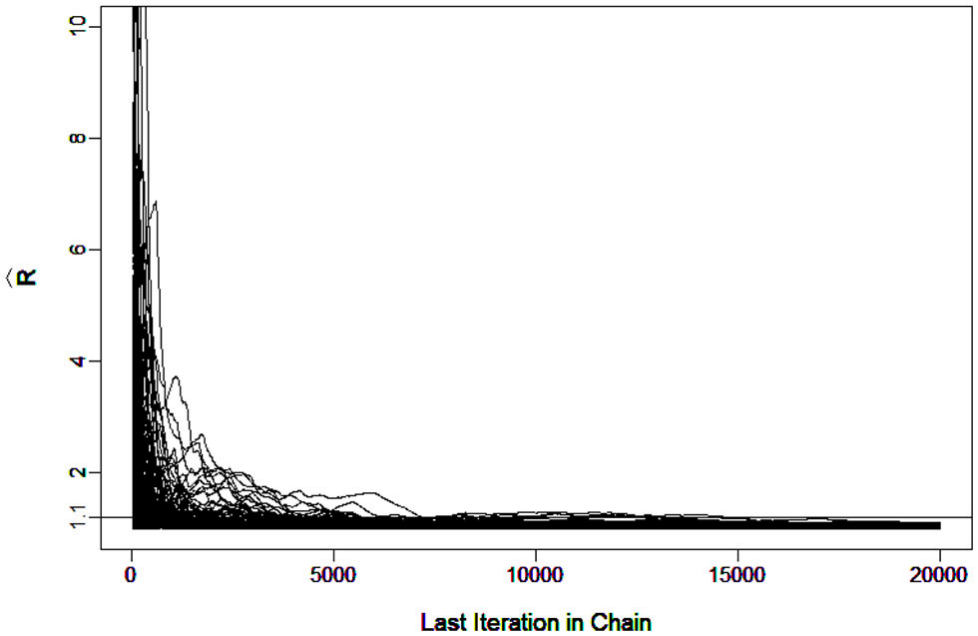
Trace Plots of $\hat{R}$ under G-DINA model for *I* = 1, 000.

It was verified that the number of iterations and burn-in were sufficient for the chain to converge. For example, Figure 3 shows the $\hat{R}$ in G-DINA model for sample size *I* = 1000 that all the parameters came down to 1.1 at the 7266th iteration.

Table 3 shows the parameter recovery results of the slice-within-Gibbs sampler under the DINA model, Table 4 shows the parameter recovery under the condition with negatively correlated item parameters, and Tables 5, 6 the results under the G-DINA model across different sample sizes and item qualities.

**Table 3:** Bias and RMSE for *s*, *g*, and π estimates under the DINA model.

	**True**			**Bias**			**RMSE**		
**I**	**s**	**g**	**π**	**s**	**g**	**π**	**s**	**g**	**π**
500	0.100	0.100	0.031	0.008	0.004	0.000	0.029	0.017	0.008
	0.200	0.100	0.031	0.011	0.002	0.000	0.033	0.023	0.009
	0.100	0.200	0.031	0.006	0.004	0.000	0.038	0.018	0.009
	0.200	0.200	0.031	0.009	0.002	0.000	0.044	0.024	0.010
1,000	0.100	0.100	0.031	0.003	0.001	0.000	0.022	0.012	0.006
	0.200	0.100	0.031	0.005	0.001	0.000	0.023	0.016	0.006
	0.100	0.200	0.031	0.004	0.002	0.000	0.030	0.013	0.006
	0.200	0.200	0.031	0.003	0.001	0.000	0.031	0.017	0.007
3,000	0.100	0.100	0.031	0.001	0.000	0.000	0.012	0.007	0.003
	0.200	0.100	0.031	0.003	0.000	0.000	0.013	0.010	0.004
	0.100	0.200	0.031	0.001	0.000	0.000	0.017	0.008	0.004
	0.200	0.200	0.031	0.001	0.000	0.000	0.019	0.011	0.005

**Table 4:** Bias and RMSE for *s*, *g* and π estimates under the negatively correlated DINA model parameters.

	**Bias**			**RMSE**		
**I**	**s**	**g**	**π**	**s**	**g**	**π**
500	0.009	0.002	0.000	0.028	0.014	0.008
1,000	0.004	0.002	−0.000	0.018	0.011	0.006
3,000	0.002	0.000	0.000	0.011	0.006	0.003

**Table 5:** Bias of ***δ*** and ***π*** estimates under the G-DINA model.

**I**	${\text{K}}_{j}^{*}$	**Intercept**	**One-way**	**Two-way**	**Three-way**	**π**
500	1	0.007	−0.006	—	—	
	2	0.008	−0.001	-0.007	—	0.000
	3	0.017	0.021	−0.044	0.053	
1,000	1	0.003	−0.003	—	—	
	2	0.003	−0.000	−0.004	—	0.000
	3	0.008	0.006	−0.016	0.020	
3,000	1	0.001	−0.001	—	—	
	2	0.001	0.000	−0.001	—	0.000
	3	0.003	−0.001	0.000	−0.001	

**Table 6:** RMSE of ***δ*** and ***π*** estimates under the G-DINA model.

**I**	${\text{K}}_{j}^{*}$	**Intercept**	**One-way**	**Two-way**	**Three-way**	**π**
500	1	0.022	0.035	—	—	
	2	0.039	0.057	0.084	—	0.015
	3	0.059	0.070	0.115	0.182	
1,000	1	0.015	0.024	—	—	
	2	0.028	0.042	0.061	—	0.012
	3	0.040	0.051	0.080	0.122	
3,000	1	0.009	0.015	—	—	
	2	0.016	0.025	0.037	—	0.006
	3	0.023	0.034	0.051	0.074	

For the smallest sample size (i.e., *I* = 500), the maximum absolute bias of the item parameter estimates was 0.011 and 0.053 for the DINA and G-DINA models; the RMSE was below 0.044 and 0.182 for the DINA and G-DINA models, respectively. With the exception of the higher-order interaction terms when $K_{j}^{*}=3$, these results indicate that satisfactory estimates can be obtained for the DINA and G-DINA models using the slice-within-Gibbs sampler even with sample size as small as *I* = 500. As the table shows, the performance of the slice-within-Gibbs sampler improved as the number of examinees increased. When *I* = 3, 000, the absolute bias and RMSE of all the item parameters were smaller, and their maximum values dropped to 0.003 and 0.019, respectively, for the DINA model, and to 0.003 and 0.074, respectively, for the G-DINA model. For the condition where the item parameters were negatively correlated, the average bias and RMSE were comparable to those obtained under the low-noise level condition. Finally, for the latent membership probabilities, all the parameters can be estimated extremely accurately (i.e., bias is 0.00) for both models. Moreover, the maximum RMSEs at *I* = 500 were 0.010 and 0.015 for the DINA and G-DINA models, respectively, and improved with larger sample sizes.

To better understand the properties of the slice-within-Gibbs sampler, Figures 1, 2 show the detailed results for *I* = 500 size under DINA model. Consistent with the results in Culpepper (2015) and de la Torre (2009), which were obtained using different estimation algorithms, worse results were obtained for items that required more attributes. The deterioration in the quality of item parameter estimates as the number of required attributes increased can be clearly observed in Figure 2, which displays the RMSE of the estimates. It should be noted that the guessing parameter estimates did in fact slightly improve with more required attributes; however, the improvement did not compensate for the stark deterioration in the slip parameter estimates. These results underscore that fact that, given a fixed same sample size, the quality of item parameter estimates of the DINA model can affected by of the number of required attributes. Finally, Figures 1, 2 indicate that item quality had only a small impact on the recovery on the individual latent class membership probabilities.

In sum, the results of Simulation Study 1 indicates that the slice-within-Gibbs sampler can provide accurate estimates of the DINA and G-DINA model parameter estimates. Moreover, it can provide results consistent with those of previously implemented algorithms.

### 4.2. Simulation Study 2

This simulation study had two-fold goals: (1) to compare the efficiency of the slice-within-Gibbs sampler to that of MH algorithm; and (2) to compare the slice-within-Gibbs sampler and Gibbs sampler in terms their flexibility in specifying the priors. For this study, the MH algorithm, Gibbs sampling and slice sampler were compared in the context of DINA model.

#### 4.2.1. Design

The simulated data contained *I* = 500 examinees, *J* = 30 items and *K* = 5 attributes. All the slip and guessing parameters were set to 0.1, and the Q-matrix given in Figure 1 was used.

For the MH algorithm, there exist infinite choices of proposal distributions. For demonstration purposes, this simulation study only considered the following two cases of the proposal distributions.

- Case 1: A larger step between iterations, where $s_{j}\sim N\left(s_{j}^{\left(t\right)},1\right)$, and $g_{j}\sim N\left(g_{j}^{\left(t\right)},1\right);$ and
- Case 2: A smaller step between iterations, where $s_{j}\sim N\left(s_{j}^{\left(t\right)},0.001\right)$, a $g_{j}\sim N\left(g_{j}^{\left(t\right)},0.001\right)$.
  For the Gibbs sampling, the Beta family distributions were the conjugate priors of the items parameters. Following Culpepper (2015), only the conjugate prior Beta(1, 1) was considered.
  For the slice-within-Gibbs sampler, both conjugate and non-conjugate priors were considered. Below are the two cases of the priors and their specific instances.
- Case 3: For conjugate priors, Beta(1, 1), Beta(1, 2) and Beta(2, 2) were used; and
- Case 4: For non-conjugate priors, *N*(0, 1)*I*_(0, 1)_(*x*), *N*(2, 1)*I*_(0, 1)_(*x*), Uniform(0, 2)*I*_(0, 1)_(*x*), and Exp(1)*I*_(0, 1)_(*x*) were used.

As in Simulation Study 1, bias and RMSE were calculated to evaluate the quality of the parameter estimates. Similarly the PSRF was computed to evaluate convergence.

#### 4.2.2. Results

Table 7 presents the recovery results of the slice-within-Gibbs sampler with uniform prior and MH algorithm under Cases 1 and Case 2. The results show that the accuracy of the MH algorithm parameter estimates was greatly influenced by the variance of proposal distribution. Specifically, the parameter estimates under Case 2 were worse than those under Case 1, which indicates that, for this particular condition, a smaller step between iterations was not a good as a larger step. It is also noteworthy that, despite the use of a uniform prior, the slice-within-Gibbs sampler provided estimates that were as good as, if not better than estimates obtained using the MH algorithm under Case 1.

**Table 7:** Bias and RMSE of the slice-within-Gibbs sampler and MH algorithm.

	**Slice sampler**		**MH - Case 1**		**MH - case 2**	
	**Bias**	**RMSE**	**Bias**	**RMSE**	**Bias**	**RMSE**
*s*	0.005	0.027	0.006	0.026	0.013	0.035
*g*	0.002	0.016	0.004	0.018	0.001	0.020
π	0.000	0.008	0.000	0.008	0.000	0.008

**Figure 4:**
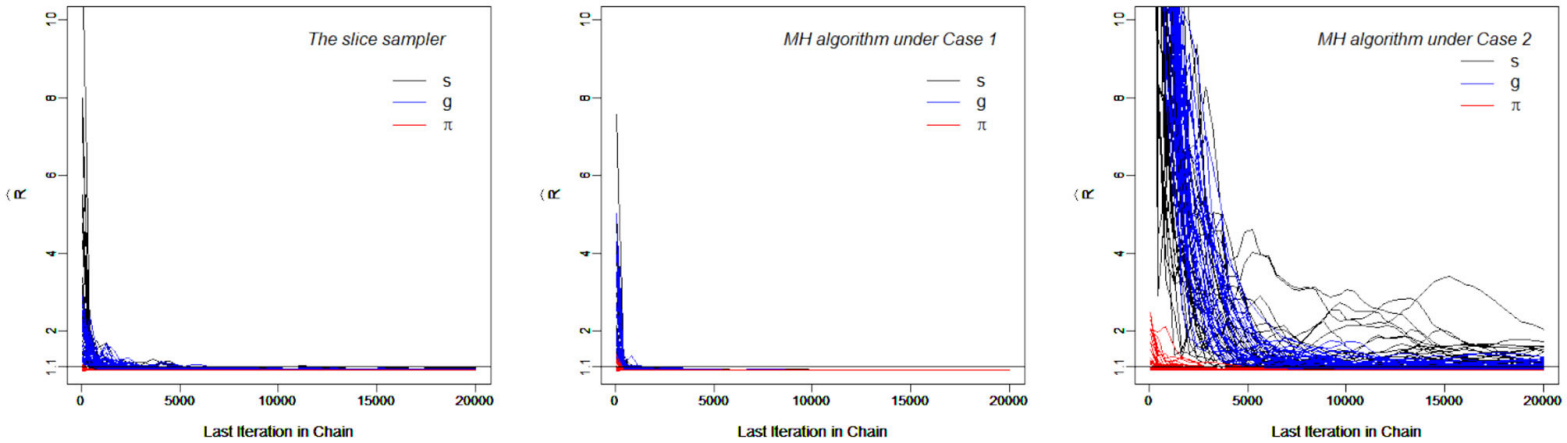
The trace Plots of $\hat{R}$ for the slice-within-Gibbs sampler and MH algorithms in Simulation Study 2.

Figure 4, which contains the $\hat{R}$s for the slice-within-Gibbs sampler and MH algorithms across different iterations, shows the differing convergence rates of the two methods. As can be seen from the figure, ***π*** converged at the fastest rate, followed by *g*. For the MH algorithm, Case 1 converged faster than Case 2 - Case 1 reached convergence by the 1000th iteration, whereas Case 2 did not even reach convergence for some parameters. This indicates that the variance of the proposal distribution in Case 2 was too small to sufficiently explore the posterior distribution. In comparison, all the parameters estimated using the slice-within-Gibbs sampler reached convergence by the 2000th iteration.

**Figure 5:**
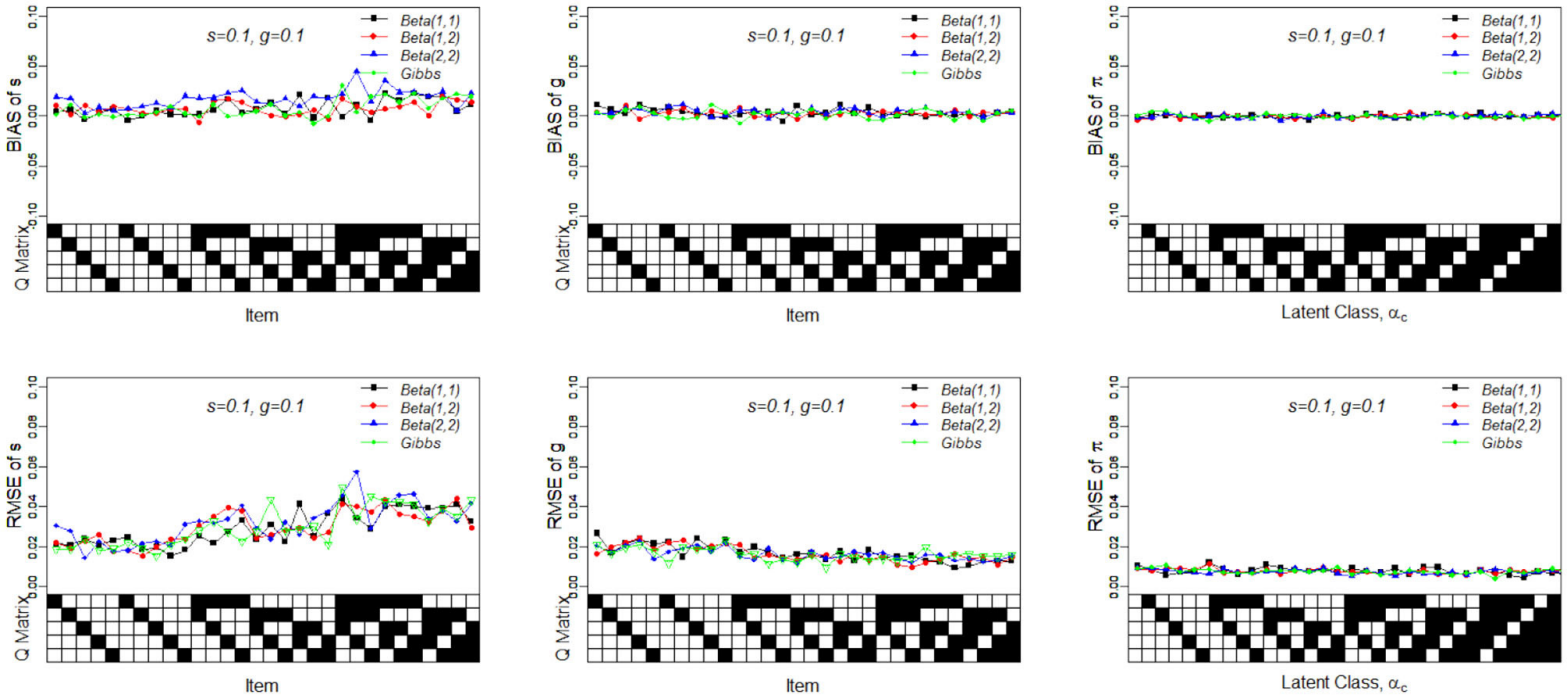
Bias and RMSE of slip, guessing, and the latent membership parameters based on different conjugate priors. Given on the X-axis are the Q-matrix and ***α***_***c***_, where a black square denotes the presence of the attribute.

Table 8 presents the recovery results of the slice-within-Gibbs sampler with Beta(1, 2) prior under Case 3 and *N*(0, 1)*I*_(0, 1)_(*x*) prior under Case 4. Figure 5 shows the bias and RMSE of the slice-within-Gibbs sampler under Case 3 (i.e., conjugate priors) and the Gibbs sampler under Beta(1, 1). It can be seen that the slice-within-Gibbs sampler performed similarly to the Gibbs sampler, particularly for *g*_*j*_ and π_*c*_. Although the estimates of *s*_*j*_ had a larger variability across the four priors, none of them was uniformly the best across the 30 items. The figure also shows that the slice-within-Gibbs sampler provided comparable results under the family of beta priors. Finally, regardless of the Beta priors used, the bias and RMSE of *s*_*j*_ were always higher than those of *g*_*j*_ and π_*c*_, which is consistent with the previous results.

**Table 8:** Bias and RMSE of the slice-within-Gibbs sampler and Gibbs algorithm.

	**Slice sampler - Case 3**		**Slice sampler - Case 4**		**Gibbs**	
	**Bias**	**RMSE**	**Bias**	**RMSE**	**Bias**	**RMSE**
*s*	0.009	0.031	0.008	0.030	0.009	0.031
*g*	0.002	0.017	0.002	0.017	0.002	0.017
π	−0.000	0.008	0.000	0.008	0.000	0.008

**Figure 6:**
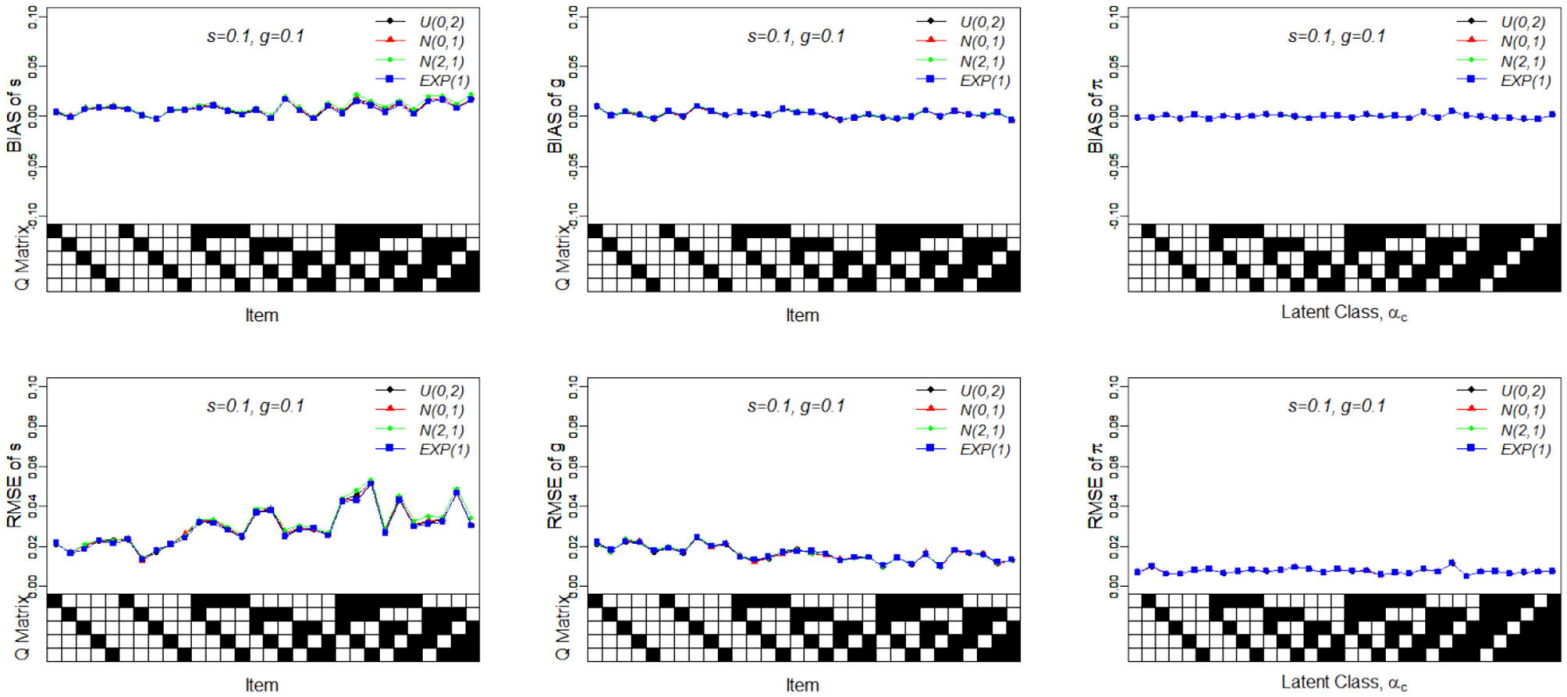
Bias and RMSE of slip, guessing and the latent membership parameters based on different non-conjugate priors. Given on the X-axis are the Q-matrix and ***α***_***c***_, where a black square denotes the presence of the attribute.

Figure 6 presents the recovery of the slice-within-Gibbs sampler under Case 4. It should be noted that the Gibbs sampler does not work under these specific priors. In contrast, the slice-within-Gibbs sampler can also be applied with different non-conjugate, even misspecified priors. The figures shows that the biases of *s*_*j*_, *g*_*j*_, and π_*c*_ were close to zero, and the corresponding RMSEs were below 0.05. Despite the use of non-conjugate priors, these results were almost the same those obtained using the Gibbs sampler under Beta(1, 1).

Table 9 compares the convergence rate of the Gibbs and slice-within-Gibbs samplers. Specifically, the simulated data based on the DINA model used *I* = 500 examinees, *J* = 30 items, *K* = 5 attributes and the Q-matrix in Figure 1. For comparison purposes, two criteria were used to evaluate the convergence rates, namely, the iterations at which all the parameters reached convergence and the time to reach 20,000 iterations. Based on 100 replications, **Table 11** shows that the Gibbs sampler converged much earlier and was about 1.29 times faster than the slice-within-Gibbs sampler.

**Table 9:** Convergence rates of the Gibbs and slice samplers.

	**Convergence observed**	
**Sampler**	**Iterations**	**Time (min)**
Slice	1,573	6.006
Gibbs	258	4.654

Overall, the results of Simulation Study 2 indicate that, depending on the proposal distribution, the slice-within-Gibbs sampler can be dramatically more or slightly less efficient than the MH algorithm. However, the MH algorithm is advantageous only to the extent that the proposal distribution is optimal, whereas the slice-within-Gibbs sampler can be implemented with a wide range of prior distributions. Similarly, although the slice and Gibbs samplers are comparable, the former, unlike the latter, is not restricted to the use of conjugate priors.

## 5. Empirical Example

### 5.1. Data

The empirical example involved fraction subtraction data previously analyzed by Tatsuoka (1990), Tatsuoka (2002) and de la Torre (2009). The data analyzed here consisted of responses of 536 students to 15 fraction subtraction items. The five attributes measured by the test were: α_1_ subtracting basic fractions; α_2_ reducing and simplifying; α_3_ separating whole from fraction; α_4_ borrowing one from whole; and α_5_ converting whole to fraction. The corresponding Q-matrix is given in Table 10.

**Table 10:** The Q-matrix of the fraction subtraction data.

**Item**	**α_1_**	**α_2_**	**α_3_**	**α_4_**	**α_5_**
1	1	0	0	0	0
2	1	1	1	1	0
3	1	0	0	0	0
4	1	1	1	1	1
5	0	0	1	0	0
6	1	1	1	1	0
7	1	1	1	1	0
8	1	1	0	0	0
9	1	0	1	0	0
10	1	0	1	1	1
11	1	0	1	0	0
12	1	0	1	1	0
13	1	1	1	1	0
14	1	1	1	1	1
15	1	1	1	1	0

### 5.2. Methods and Results

**Figure 7:**
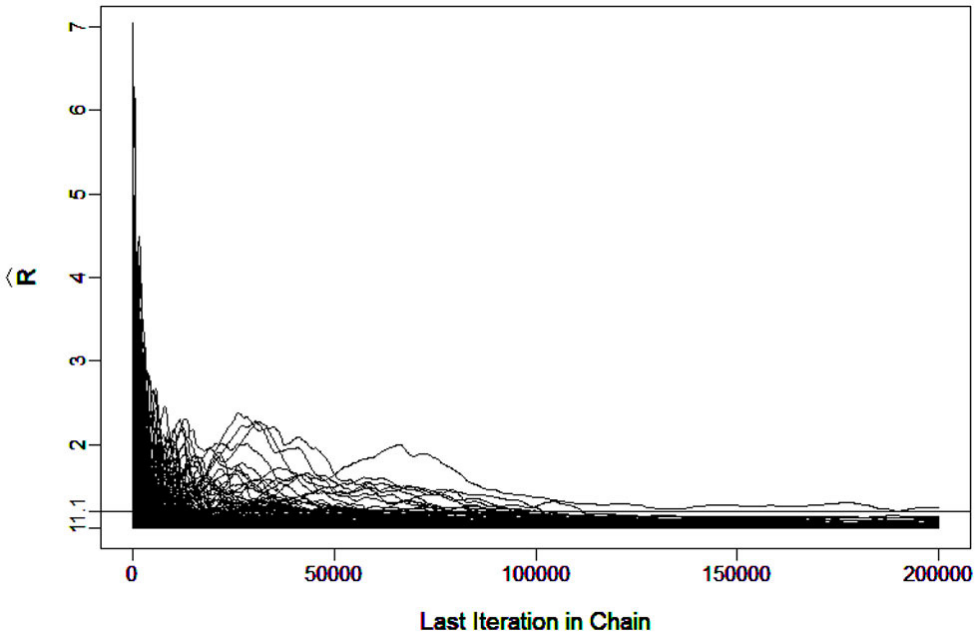
Trace Plots of $\hat{R}$ for the real data in the GDINA model.

**Figure 8:**
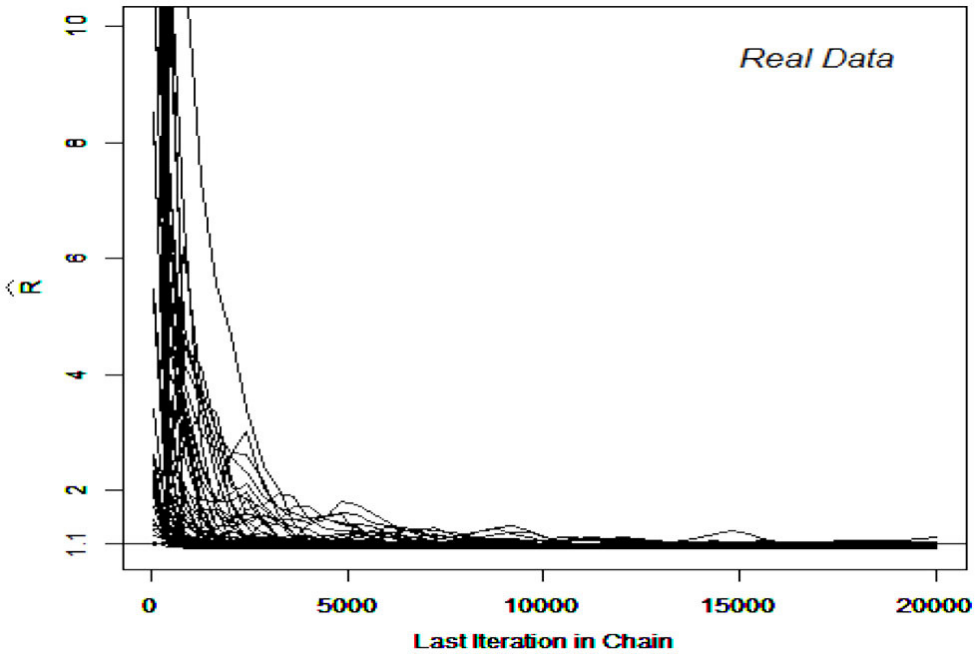
Trace Plots of $\hat{R}$ for the real data in the DINA model.

The DINA and G-DINA models were fitted to the data and the corresponding parameters estimated using the slice-within-Gibbs sampler, with monotonicity constraints imposed to stabilize the estimates due to the relatively small sample size. Incidentally, the Gibbs sampler was not considered for these data due to the difficulty in finding conjugate priors that can also accommodate the monotonicity constraints. The estimates based on the expected a posteriori (EAP) and the corresponding standard errors (SEs) were computed for DINA and G-DINA models. Finally, the deviance information criterion (DIC) was employed to select between the two models. Figures 7, 8 show the $\hat{R}$ for the G-DINA and DINA analyses of the empirical data, respectively. In addition to the convergence of the chains, the figures also show that the DINA model converged faster than the G-DINA model for these data.

In terms of DIC, a model with smaller DIC is to be preferred (Spiegelhalter et al., 2002). In fitting the fraction subtraction data, the DICs of the DINA and G-DINA models were 27719.86 and 27017.43, respectively, which indicates that the G-DINA model provided a better fit to data. Thus, only results pertaining to the G-DINA model are presented below.

Table 11 contains the EAP estimates of the latent membership parameters, ${\hat{\pi }}_{c}$, and their corresponding SEs under the G-DINA model. The eight latent classes with the largest memberships were: π(1, 1, 1, 1, 1) = 0.335, π(1, 1, 1, 0, 0) = 0.138, π(1, 1, 1, 1, 0) = 0.118, π(0, 0, 0, 1, 0) = 0.110, π(1, 1, 1, 0, 1) = 0.083, π(0, 0, 1, 0, 0) = 0.079, π(1, 0, 1, 0, 0) = 0.035, and π(1, 1, 0, 1, 0) = 0.014. They accounted for over 91% of the latent class memberships. In terms of individual attribute mastery, α_1_ through α_5_ had the following prevalences: 0.771, 0.723, 0.812, 0.620, and 0.463, which makes α_3_ and α_5_ the easiest and most difficult attributes to master, respectively. It can be noted that latent classes which showed mastery of all but one of the three easiest attributes to master, as in (0,1,1,1,1), (1,0,1,1,1), and (1,1,0,1,1), had the lowest latent class memberships. In this example, it can be noted that latent classes with the largest class memberships also had the larger SEs.

**Table 11:** The EAP of the latent class parameters under G-DINA model.

**Latent classes**					$\hat{\pi }$		**Latent classes**					$\hat{\pi }$	
**α_1_**	**α_2_**	**α_3_**	**α_4_**	**α_5_**	**EAP**	**SE**	**α_1_**	**α_2_**	**α_3_**	**α_4_**	**α_5_**	**EAP**	**SE**
0	0	0	0	0	0.005	0.004	1	1	1	0	0	0.138	0.011
1	0	0	0	0	0.005	0.003	1	1	0	1	0	0.014	0.006
0	1	0	0	0	0.004	0.003	1	1	0	0	1	0.003	0.003
0	0	1	0	0	0.079	0.011	1	0	1	1	0	0.001	0.003
0	0	0	1	0	0.110	0.010	1	0	1	0	1	0.006	0.003
0	0	0	0	1	0.004	0.004	1	0	0	1	1	0.013	0.005
1	1	0	0	0	0.006	0.004	0	1	1	1	0	0.003	0.002
1	0	1	0	0	0.035	0.007	0	1	1	0	1	0.001	0.001
1	0	0	1	0	0.009	0.006	0	1	0	1	1	0.006	0.001
1	0	0	0	1	0.003	0.002	0	0	1	1	1	0.001	0.001
0	1	1	0	0	0.006	0.006	1	1	1	1	0	0.118	0.013
0	1	0	1	0	0.002	0.002	1	1	1	0	1	0.083	0.008
0	1	0	0	1	0.002	0.002	1	1	0	1	1	0.001	0.001
0	0	1	1	0	0.003	0.002	1	0	1	1	1	0.001	0.001
0	0	1	0	1	0.001	0.001	0	1	1	1	1	0.001	0.001
0	0	0	1	1	0.002	0.002	1	1	1	1	1	0.335	0.015

Table 12 gives the G-DINA model estimates of the fraction subtraction items in term of the latent group success probability $P\left(\alpha_{ij}^{*}\right)$. The item parameter estimates clearly show why the G-DINA model was preferred over the DINA model. For the DINA model to provide a satisfactory fit to the data, all parameters and except the intercept and the highest-order interaction effect must be equal to zero. This was not the case with, say, items that require two attributes (i.e., items 8, 9, and 12). For these items, *P*(00) < *P*(10) and *P*(00) < *P*(01) indicating that the main effects are not equal to zero. The remaining multi-attribute items also indicate that the conjunctive assumption of the DINA model was not tenable. As a rough measure of item discrimination, Δ_*j*_ = *P*_*j*_(**1**) − *P*_*j*_(**0**), was computed. All but two items had Δ_*j*_ > 0.70, and the average item discrimination was ${\bar{\Delta }}_{j}=0.829$. These results indicate that the fraction subtraction items are highly discriminating.

**Table 12:** Results of the fraction subtraction data analysis under G-DINA model using the slice-within-Gibbs sampler and EM algorithm.

$\text{P}\left(\alpha_{ij}^{*}\right)$																	
		0	1														
		00	10	01	11												
		000	100	010	001	110	101	011	111								
		0000	1000	0100	0010	0001	1100	1010	1001	0110	0101	0011	1110	1011	1101	0111	1111
		00000	10000	01000	00100	00010	00001	11000	10100	10010	10001	01100	01010	01001	00110	00101	00011
Method	Item	11100	11010	11001	10110	10101	10011	01110	01101	01011	00111	11110	11101	11011	10111	01111	11111
	1	0.103	0.877														
	2	0.029	0.170	0.054	0.064	0.353	0.301	0.530	0.527	0.110	0.426	0.496	0.655	0.650	0.823	0.632	0.922
	3	0.511	0.969														
	4	0.003	0.026	0.008	0.009	0.017	0.069	0.064	0.148	0.058	0.400	0.025	0.045	0.151	0.072	0.140	0.144
		0.315	0.126	0.533	0.277	0.513	0.484	0.189	0.291	0.280	0.275	0.549	0.718	0.674	0.673	0.498	0.939
	5	0.370	0.853														
	6	0.003	0.056	0.009	0.009	0.037	0.237	0.217	0.409	0.024	0.167	0.156	0.425	0.676	0.630	0.353	0.840
	7	0.011	0.043	0.036	0.045	0.073	0.118	0.453	0.643	0.099	0.155	0.328	0.547	0.723	0.908	0.419	0.963
	8	0.119	0.823	0.857	0.957												
The slice	9	0.060	0.904	0.755	0.964												
sampler	10	0.006	0.097	0.027	0.031	0.147	0.332	0.202	0.561	0.326	0.335	0.253	0.716	0.736	0.700	0.584	0.952
	11	0.055	0.828	0.757	0.940												
	12	0.006	0.060	0.061	0.023	0.236	0.662	0.735	0.935								
	13	0.006	0.324	0.047	0.046	0.023	0.451	0.494	0.657	0.110	0.204	0.254	0.605	0.747	0.783	0.388	0.863
	14	0.001	0.012	0.006	0.006	0.007	0.011	0.050	0.058	0.046	0.069	0.017	0.042	0.056	0.051	0.060	0.063
		0.182	0.130	0.213	0.174	0.227	0.268	0.166	0.186	0.221	0.220	0.433	0.457	0.523	0.564	0.474	0.891
	15	0.002	0.011	0.012	0.009	0.017	0.074	0.067	0.301	0.032	0.354	0.324	0.278	0.660	0.677	0.597	0.910														
	1	0.088	0.880
	2	0.000	0.000	0.000	0.096	0.298	0.000	0.419	0.811	0.096	0.298	0.318	0.433	0.871	0.811	0.366	0.908
	3	0.513	0.970														
	4	0.000	0.000	0.000	0.000	0.031	0.000	0.014	0.000	0.031	0.235	0.000	0.031	0.000	0.031	0.000	0.031
		0.587	0.031	0.819	0.319	0.396	0.908	0.031	0.000	0.345	0.031	0.908	0.823	0.908	0.908	0.346	0.908
	5	0.327	0.839														
	6	0.000	0.000	0.000	0.000	0.025	0.100	0.104	0.025	0.000	0.025	0.025	0.300	0.641	0.833	0.025	0.833
	7	0.000	0.000	0.000	0.019	0.051	0.000	0.019	0.335	0.110	0.066	0.051	0.586	0.972	0.768	0.332	0.972
	8	0.000	0.853	0.953	0.953												
EM	9	0.032	0.912	0.721	0.971												
algorithm	10	0.000	0.000	0.000	0.000	0.000	0.000	0.168	0.751	0.215	0.000	0.945	0.712	0.945	0.945	0.945	0.945
	11	0.011	0.783	0.770	0.952												
	12	0.000	0.052	0.039	0.000	0.052	0.641	0.930	0.930								
	13	0.000	0.000	0.054	0.047	0.000	0.647	0.248	0.362	0.090	0.066	0.047	0.857	0.837	0.857	0.457	0.857
	14	0.000	0.000	0.000	0.000	0.000	0.000	0.000	0.000	0.000	0.000	0.000	0.000	0.000	0.000	0.000	0.000
		0.265	0.098	0.000	0.000	0.301	0.245	0.000	0.000	0.000	0.000	0.879	0.740	0.245	0.497	0.001	0.879
	15	0.000	0.000	0.000	0.000	0.000	0.000	0.000	0.000	0.000	0.122	0.000	0.000	0.704	0.630	0.914	0.914

**Figure 9:**
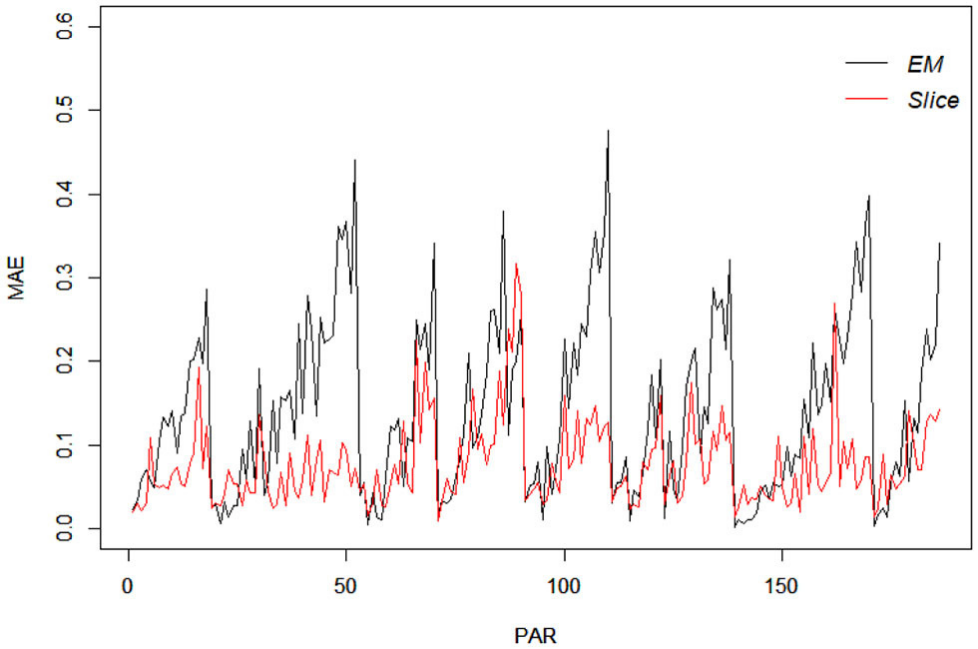
Mean absolute bias of the slice-within-Gibbs sampler and EM estimates of data simulated based on the fraction subtraction data.

For comparison purpo ses, the EM estimates of the same items were also obtained using the R package “GDINA” (Ma and de la Torre, 2020), and are given Table 12. It can be noted that for one-attribute items (i.e., items 1 and 5), the slice-within-Gibbs sampler and EM estimates are highly comparable. However, for multi-attribute items, the estimates can be quite disparate, except for *P*_*j*_(**1**) was comparable across the two methods. The difference could be due to the small sample size relative to the complexity of the G-DINA model for the multi–attribute items. To better understand the behavior of the slice-within-Gibbs sampler and EM algorithm vis-a-vis the fraction subtraction data, we conducted a simulation study where data were generated based on the parameters obtained using the slice-within-Gibbs sampler. Figure 9 shows the mean absolute error (MAE) of the two estimates across 100 replications. The figure indicates that the slice-within-Gibbs sampler had smaller mean absolute biases for most of the parameters, thus, a more reliable method for the fraction subtraction data.

## 6. Discussion

In this work, the slice-within-Gibbs sampler is introduced as a method of estimating CDMs. Unlike the MH algorithm, the slice-within-Gibbs sampler obviates the need to choosing an optimal proposal distribution; unlike Gibbs sampler, the slice-within-Gibbs sampler has the flexibility to work with a wider range of prior distributions. As shown in the simulation studies and empirical example, it can be used to estimate complex CDMs, such as the G-DINA model. Thus, the slice-within-Gibbs sampler provides an alternative and viable estimation procedure in the context of CDMs.

Based on the results of Table 9, additional work is needed to speed up the implementation of the slice-within-Gibbs sampler for researchers to be able to fully take advantage of the flexibility of the sampler to estimate a wide range CDMs.

In the present work, only two CDMs (i.e., DINA and G-DINA models) were employed to illustrate the slice-within-Gibbs sampler. However, the slice-within-Gibbs sampler can be easily extended to other CDMs (e.g., additive CDM, GDM), attribute structure (e.g., higher-order CDMs; de la Torre and Douglas, 2004), and potentially to CDMs that incorporate various types of covariates.

Finally, it should be noted that other MCMC sampling procedures that use auxiliary variables are currently available. One such procedure is the Hamiltonian Monte Carlo (Neal, 2011; Duane et al., 1987, HMC) algorithm. The HMC algorithm is based on the *Hamiltonian dynamics*, and has a physical interpretation and can provide useful intuitions. As an extension of the MH algorithm, it exploits the gradient information to draw samples from the posterior. Because HMC algorithm often provides a large move with acceptance rates close to one, its efficiency is higher than that of the MH algorithm. Future research should systematically compare the performance of the slice-within-Gibbs sampler and the HMC algorithm, as well as other auxiliary-variable sampling procedures, in estimating CDMs.

## Data Availability Statement

Publicly available datasets were analyzed in this study. This data can be found here: https://CRAN.R-project.org/package=CDM.

## Ethics Statement

Written informed consent was obtained from the individual(s), and minor(s)' legal guardian/next of kin, for the publication of any potentially identifiable images or data included in this article.

## Author Contributions

XX and JG worked on the technical details of the article. XX and JZ completed the writing, with the support of JT. JT and JZ provided a few suggestions on the focus and direction of the research. All authors contributed to the article and approved the submitted version.

## Conflict of Interest

The authors declare that the research was conducted in the absence of any commercial or financial relationships that could be construed as a potential conflict of interest.
