# Supplementary material for: Estimating CDMs Using the Slice-Within-Gibbs Sampler
Source: Front Psychol. 2020 Sep 25;11:2260. doi: 10.3389/fpsyg.2020.02260 (PMC7545134; doi:10.3389/fpsyg.2020.02260)
Supplement: Supplementary file 1 [file Data_Sheet_1.PDF]

frontiers

Supplementary Material

1 R CODE

```
set.seed(1234)
library('MCMCpack');library("CDM");library("dina");library(coda)
J <- 30;iteration <-20000;K <- 5;C <- 2^K
Rcpp::sourceCpp('updata_alpha.cpp')
q.matrix <- matrix( 0, J,K)
diag(q.matrix[1:5,]) <- 1
diag(q.matrix[6:10,]) <- 1
q.matrix[c(11:14,21:26),1] <- 1
q.matrix[c(11,15:17,21:23,27:29),2] <- 1
q.matrix[c(12,15,18,19,21,24,25,27,28,30),3] <- 1
q.matrix[c(13,16,18,20,22,24,26,27,29,30),4] <- 1
q.matrix[c(14,17,19,20,23,25,26,28:30),5] <- 1
slip <- rep(0.1,J);guess <- rep(0.1,J);
K <- ncol(q.matrix);
CC <- 2^K;
burnin <- 10000
alpha <- matrix(rep(0, K * CC), ncol = K)
h1 <- 2
if (K >= 2) {
  for (lll in 1:(K- 1)) {
    lk <- combn(1:K, lll)
    for (jjj in 1:(ncol(lk))) {
      alpha[h1, lk[, jjj]] <- 1
      h1 <- h1 + 1
    }
  }
}
alpha[CC, ] <- rep(1, K)
set.seed(1)
I<-500;alpha0 <- matrix(0,I,K);
alpha0[1:I,] <- alpha[sample(1:32,I,replace=TRUE),];
RR <- sim.din(q.matrix = q.matrix, guess=guess,slip=slip,
              rule = "DINA",alpha=alpha0);
response <- RR$dat
slice <- function(response,q.matrix,iteration,burnin){
  K <- ncol(q.matrix);
  I <- nrow(response);
  J <- ncol(response);
  CC <- 2^K;
  S <- matrix(0,nrow=iteration+1,ncol=J);
```

```
G<- matrix(0,nrow=iteration+1,ncol=J);
p<-matrix(0,nrow=iteration+1,ncol=CC)
S[1,] <-rep(runif(1,0,0.2),J);G[1,] <-rep(runif(1,0,0.2),J);
alpha <- matrix(rep(0, K * CC), ncol = K)
h1 <- 2
if (K >= 2) {
  for (lll in 1:(K- 1)) {
    lk <- combn(1:K, lll)
    for (jjj in 1:(ncol(lk))) {
      alpha[h1, lk[, jjj]] <- 1
      h1 <- h1 + 1
    }
  }
}
alpha[CC, ] <- rep(1, K)
ness.attr=rowSums(q.matrix)
p[1,] <- rep(1/CC,CC)
delta<-rep(1,CC);A<-alpha[sample(1:CC,I,replace=TRUE),];
for(t in 1:iteration){
  update_a<-update_alpha(Amat=alpha,Q=q.matrix,ss=S[t,],
  gs=G[t,],Y=response,PIs=p[t,],ALPHAS=A,delta0=delta)
  p[t+1,]<-update_a$PIs_new
  cc <- update_a$CLASSES+1
  A<-alpha[cc,]
  poss.attr <- A%*%t(q.matrix)
  eta.pp=poss.attr>=outer(rep(1,I),ness.attr)
  ETA= 1*(eta.pp=="TRUE")+0*(eta.pp=="FALSE")
  U1<-runif(I*J,0,t(outer(1-S[t,],rep(1,I))));
  U2<-runif(I*J,0,t(outer(S[t,],rep(1,I))));
  P1 <- 1-U1*response*ETA;P2<-U2*(1-response)*ETA
  c <- apply(P1,2,min)
  s_right <- apply(rbind(c,1-G[t,]),2,min)
  s_left <- apply(P2,2,max)
  S[t+1,] <- runif(J,s_left,s_right)
  V1<-runif(I*J,0,t(outer(1-G[t,],rep(1,I))));
  V2<-runif(I*J,0,t(outer(G[t,],rep(1,I))));
  P1 <- 1-V1*(1-response)*(1-ETA);P2<-V2*response*(1-ETA)
  c <- apply(P1,2,min)
  g_right <- apply(rbind(c,1-S[t+1,]),2,min)
  g_left <- apply(P2,2,max)
  G[t+1,] <- runif(J,g_left,g_right)
}

item_est <- matrix(0,2,J)
item_est[1,]<- c(colMeans(S[burnin:iteration,]))
```

```
  item_est[2,]<- c(colMeans(G[burnin:iteration,]))
  rownames(item_est)<-c("slip","guessing")
  colnames(item_est) <- 1:J
  pi_est<- colMeans(p[burnin:iteration,])
  names(pi_est) <- 1:CC
  par_est <- list(item_est,pi_est)
  names(par_est) <- c("item_est", "pi_est")
  return(par_est)
}
set.seed(1234)
result <- slice(response,q.matrix,iteration,burnin)
item_result <- result$item_est;
pi_result <- result$pi_est;
```

```
// [[Rcpp::depends(RcppArmadillo,rgen,simcdm)]]
#include <RcppArmadillo.h>
#include <rgen.h>
#include <simcdm.h>

// [[Rcpp::export]]
Rcpp::List update_alpha(const arma::mat &Amat,
                        const arma::mat &Q,
                        const arma::vec &ss,
                        const arma::vec &gs,
                        const arma::mat &Y,
                        const arma::vec &PIs,
                        arma::mat &ALPHAS,
                        const arma::vec &delta0)
{

  unsigned int N = Y.n_rows;
  unsigned int J = Q.n_rows;
  unsigned int C = Amat.n_rows;
  unsigned int K = Q.n_cols;
  double ci;

  arma::mat alphas_new = arma::zeros<arma::mat>(N, K);
  arma::vec PYCS(C);
  arma::vec CLASSES(N);
  arma::vec PS;
  arma::vec Ncs = arma::zeros<arma::vec>(C);
  double etaij;

  for (unsigned int i = 0; i < N; i++) {
    PYCS = arma::ones<arma::vec>(C);
```

```

    for (unsigned int c = 0; c < C; c++) {

      for (unsigned int j = 0; j < J; j++) {
        etaij = 1.0;
        if (arma::dot(Amat.row(c), Q.row(j)) <
          arma::dot(Q.row(j), Q.row(j))) {
          etaij = 0.0;
        }
        if (etaij == 1.0 and Y(i, j) == 1.0) {
          PYCS(c) = (1.0 - ss(j)) * PYCS(c);
        }
        if (etaij == 0.0 and Y(i, j) == 1.0) {
          PYCS(c) = gs(j) * PYCS(c);
        }
        if (etaij == 1.0 and Y(i, j) == 0.0) {
          PYCS(c) = ss(j) * PYCS(c);
        } else {
          PYCS(c) = (1.0 - gs(j)) * PYCS(c);
        }
      }
    }
    PS = PYCS % PIs
    / (arma::conv_to<double>::from(PYCS.t() * PIs));
    ci = rgen::rmultinomial(PS);
    ALPHAS.row(i) = Amat.row(ci);
    Ncs(ci) = 1.0 + Ncs(ci);
    CLASSES(i) = ci;
  }
  return Rcpp::List::create(
    Rcpp::Named("PYCS") = PYCS, Rcpp::Named("PS") = PS,
    Rcpp::Named("PIs_new") = rgen::rdirichlet(Ncs + delta0),
    Rcpp::Named("CLASSES") = CLASSES);
}
```
